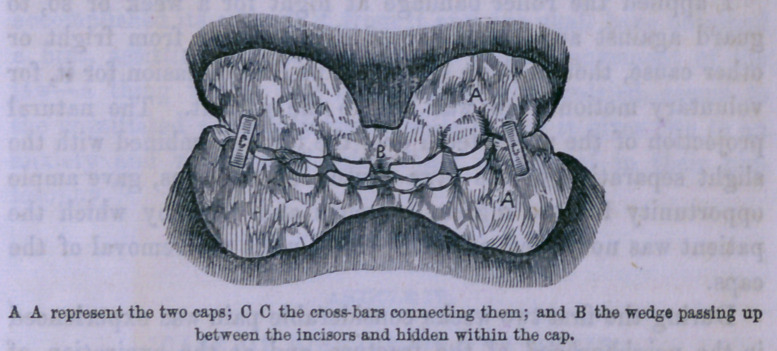# Case of Fracture of the Lower Jaw at Its Neck

**Published:** 1858-08

**Authors:** M. O. Heydock

**Affiliations:** Chicago, Ill.


ARTICLE III.

### A CASE OF FRACTURE OF THE LOWER JAW AT ITS NECK.

BY M. O. HEYDOCK, M. D., CHICAGO, ILL.

I was called upon the evening of the 21st of May to see Mrs. B. F. H., who had, I was told, just been thrown from her carriage and severely injured. Upon my arrival, I found that a fracture of the lower jaw constituted the only injury of a serious nature; the treatment of which is the subject of this paper.

The fracture was evidently at the base of the left condyle, crepitus was very marked and distinct, and deformity arose from a swaying of the jaw to the left, owing, as I suppose, to contraction of the pterygoid muscle. The fracture was easily reduced, the ordinary roller bandage applied, and cold water dressings ordered for the night.

Upon calling the next morning, I found that soon after falling asleep, displacement had taken place, and it was in the same condition as at first.

I now applied a pasteboard mould and the roller as securely as possible. The next morning I found that this had served the purpose during the day, yet during sleep displacement had again occurred, as in the previous night.

I now, with the approval of Dr. Freer, who had seen the case with me, had a spring made, partially encircling the neck, having a pad at each extremity, making pressure upon the left ramus and over the right articulation, hoping to counteract and overcome the action of the pterygoid muscle.

This acted indifferently well and was thrown aside. I now used one after another, starch binders, board and straps, and during the ensuing ten days almost every bandage I could find, approved by authors or suggested by friends. But each and every one failed me in my effort to retain the jaw in its place during the night, when voluntary control was lost in slumber. Two weeks had elapsed, displacement had occurred each and every night, crepitus was still as marked as ever, and pain on motion as great.

The prospect was anything but encouraging, a false joint seemed not only a possibility but a probability, unless by some contrivance immobility could soon be obtained.

I had exhausted my inventive resources in external appliances, and I now made a careful examination of the teeth to see if from them I could not obtain some hint which would assist me to accomplish the object I had in view. I observed that the upper central incisors were widely separated, and it occurred to me that advantage might in some way be taken of this peculiarity. A few hours elaborated the idea which had presented itself, and which was with the modifications to be hereafter mentioned, successfully carried out and executed.

The idea suggested was that a mould of the teeth of the lower jaw should be taken, running as far back as the molars, and from this a gold cap should be made which should snugly fit, and be securely and firmly attached to the teeth by clasps or otherwise. To this cap, at the point of separation between the central incisors spoken of above, a fragment of gold was to be attached, which should, when the jaws were closed, pass up between these incisors somewhat in the manner of a wedge.

If I am understood, it will be seen that if the cap retains its place, I have by the wedge-like process overcome the tendency to lateral displacement.

I now called upon Dr. Allport, a dentist of our city, and stated my project to him. He suggested that the pressure of the wedge, even though slight, and though not exerted during the day, while the jaw was under the control of the patient, might give rise to some soreness and irritation if continued as long as the nature of the case demanded. He proposed to overcome this objection, by fitting a cap for the upper jaw, similar to that of the lower, into which the wedge should be inserted, thus distributing the pressure. The two caps were then to be secured together by a couple of slender bars crossing from one cap to the other.

A A represent the two caps; C C the cross-bars connecting them; and B the wedge passing up between the incisors and hidden within the cap.

Impressions were now taken in wax, and the caps made after the same manner as the plates made by dentists for artificial teeth.

Upon placing the caps in position, it was found as we had anticipated, that the gold operating on a foreign body would not admit of a closure of the jaws. To obviate this, the opposing and impinging surfaces were freely cut away, exposing the crowns of all the teeth, thus permitting a close approximation of the jaws, and at the same time furnishing an outlet for the secretions which would naturally accumulate within the caps, and prove a source of annoyance and irritation.

The relation one cap would bear to the other was ascertained by placing the chin in position and directing the patient to bite into a mass of softened wax, and while the caps were imbedded in the wax they were removed and secured in this relation by bars crossing in the neighborhood of the canine teeth of each side.

Dr. Allport now suggested that a thin layer of gutta percha should be placed inside the caps, which might, by its presence as a lining, prove less irritating than the plates alone, while at the same time it would more perfectly adapt itself to the inequalities of the surfaces than the metal. This, after being softened in boiling water, was placed within the caps and they placed *in situ* while it was soft and pliable and as hot as could well be borne. The patient was then directed to shut the jaw naturally, and it was then firmly pressed home and immovably fixed.

I applied the roller bandage at night for a week or so, to guard against any possibility of displacement from fright or other cause, though there seemed to be little occasion for it, for voluntary motion even was lost to the patient. The natural projection of the upper teeth over the lower, combined with the slight separation of the jaws caused by the caps, gave ample opportunity for the ingress of soups and slops by which the patient was nourished and supported, until the removal of the caps.

During the first two weeks considerable pain was experienced in the neighborhood of the fracture, and at the expiration of that time the provisional callus was perceptible through the tissues.

Four weeks having elapsed from the day in which the caps were applied, and six from the date of fracture, they were removed, and union found to be complete. For the first few days the articulating surfaces of the teeth did not readily coalesce, but at the end of a fortnight the recovery was perfect and satisfactory in every particular.

There was, upon removing the caps, some soreness of the mucous membrane, but this rapidly subsided under the use of an astringent gargle combined with chlorate of potash.

As a general rule fractures of the jaw are easily treated, the roller, pasteboard and starch proving sufficient. But in this case they were of no service in restraining laterial motion, and until this experience, I confess I was not aware how difficult a thing it was to control that movement. I certainly gave them a fair trial, for Fergusson tells us in his work on surgery that he "does not take particular pains about the bandages after the fifteenth day," while in this two weeks elapsed without *any* perceptible change for the better; nor was there any promise that there would be if the same course was continued for a longer period.

In closing, I would say, that it is not of course to be expected that in all fractures of this nature, the incisors will be found conveniently separated and admitting of an application of just such a contrivance as this. But this report will have accomplished its errand, if from it any one shall have obtained a hint, assisting him to control the lateral movement of the jaw—a thing which, like many things else untried, seems to the uninitiated simplicity itself, while in fact it gives rise to an anxiety and vexation of spirit "not dreamt of in their philosophy."

---

ARTICLE IV.

## Figures and Tables

**Figure f1:**